# Musical and Multilingual Experience Are Related to Healthy Aging: Better Some Than None But Even Better Together

**DOI:** 10.1093/geronb/gbac185

**Published:** 2022-12-01

**Authors:** Saskia Esmee Nijmeijer, Marie-José van Tol, André Aleman, Merel Keijzer

**Affiliations:** Cognitive Neuroscience Center, Department of Biomedical Sciences of Cells and Systems, University Medical Center Groningen, University of Groningen, Groningen, The Netherlands; Cognitive Neuroscience Center, Department of Biomedical Sciences of Cells and Systems, University Medical Center Groningen, University of Groningen, Groningen, The Netherlands; Cognitive Neuroscience Center, Department of Biomedical Sciences of Cells and Systems, University Medical Center Groningen, University of Groningen, Groningen, The Netherlands; Bilingualism and Aging Lab, Center for Language and Cognition Groningen, Faculty of Arts, University of Groningen, Groningen, The Netherlands

**Keywords:** Bilingualism, Cognition, Complex life experiences, Music, Well-being

## Abstract

**Objectives:**

Life experiences that are complex, sustained, and intense, such as active participation in music and speaking multiple languages, have been suggested to contribute to maintaining or improving cognitive performance and mental health. The current study focuses on whether lifetime musical and multilingual experiences differentially relate to cognition and well-being in older adults, and tests whether there is a cumulative effect of both experiences.

**Methods:**

A total of 11,335 older adults from the population-based Lifelines Cohort Study completed a musical and multilingual background and experience questionnaire. Latent class analysis was used to categorize individuals into subgroups according to their various musical and multilingual experiences resulting in a (1) nonmusical, low-multilingual group; (2) nonmusical, high-multilingual group; (3) musical, low-multilingual group; and (4) musical high-multilingual group. To determine whether the groups differed in terms of cognition or emotional affect, differences in Ruff Figural Fluency Test (RFFT) and Positive and Negative Affect Schedule scores were investigated by means of multinomial logistic regression analysis.

**Results:**

Having high-multilingual, and not musical, experience was related to better RFFT performance compared to no experience, but not to more positive affect. Having both musical and high-multilingual experiences is related to better RFFT performance and more positive affect in advanced age compared to having only one experience or none. Importantly, these results were found independently of age, level of education, and socioeconomic status.

**Discussion:**

Musical and multilingual experiences are related to healthy aging, especially when combined, which supports the suggestion that a broader spectrum of lifetime experiences relates to cognitive reserve.

Worldwide, the proportion of older adults is greatly increasing, attributable to the rapidly increasing human life expectancy (European Commission, 2020). However, a longer life does not necessarily entail a longer healthy life. Research targeting the promotion of healthy aging to preserve physical and mental health is more topical than ever before.

The older adult life stage is characterized by substantial individual variation in the extent to which older adults experience age-associated ailments. For instance, the degree of cognitive decline vastly differs, possibly due to differences in resilience and cognitive reserve, built through a lifetime of experiences related to, among many other things, education, mental stimulation, and traits such as intelligence (Song et al., 2022; Stern, 2021). Being mentally and physically active and socially integrated in life has been associated with preservation of cognitive and mental health and may act as a protective mechanism against dementia (Dause & Kirby, 2019; Fratiglioni et al., 2004), contribute to (self-perceived) quality of life (Machón et al., 2017), and in turn emotional well-being (Scheibe & Carstensen, 2010).

Cognitive maintenance in older adulthood is partly experience-based and has been most notably linked to cognitively complex, sustained, and intense life experiences (reviewed in, e.g., Kramer et al., 2004; Song et al., 2022). Two particularly prominent activities in this respect, which are commonly practiced and experienced by many people across the life span, are active participation in music (i.e., playing a musical instrument or singing in a choir) and speaking multiple languages.

Playing a musical instrument or engaging with music through singing (in a choir) is related to cognitive ability and healthy aging (reviewed in Román-Caballero et al., 2018; Schneider et al., 2019). It is a complex and sustained activity that requires a broad range of cognitive processes and has been shown to induce neuroplastic changes in brain networks underlying these processes (reviewed in Moreno & Bidelman, 2014; Olszewska et al., 2021). It has been related to improved cognition and the preservation of cognitive functioning in adulthood (Hanna-Pladdy & MacKay, 2011; Mansky et al., 2020). Furthermore, short-term musical training in older adulthood can improve cognition (Alain et al., 2019; Bugos et al., 2007), and well-being (Seinfeld et al., 2013) in healthy older adults, as well as in older adults with mild cognitive impairment (MCI; Dorris et al., 2021).

Speaking multiple languages is another intense and complex activity that is typically sustained through life and that has been related to improved cognition (Bialystok, 2017). Similar to playing a musical instrument, speaking multiple languages requires several cognitive processes. This includes monitoring, inhibition of, and switching between languages. These cognitive processes are needed to resolve the constant conflict alleged to occur by juggling multiple languages (Kroll & Bialystok, 2013). Speaking multiple languages has furthermore been linked to neuroplastic changes across the life span (reviewed in Li et al., 2014), in turn contributing to improved cognitive functioning (Bialystok, 2017; Bialystok et al., 2012). Neuroplastic changes resulting from multilingual experiences potentially protect against age-related cognitive decline (van den Noort et al., 2019; Watson et al., 2016). Also, language training in older adulthood has been related to improved well-being and cognition (Bubbico et al., 2019; Pfenninger & Polz, 2018). It should be noted, however, that multilingual practices as cognitive enhancement is sometimes disputed (reviewed in, e.g., Mukadam et al., 2017). Behavioral consequences have been repeatedly shown not to be present, even in the case of neural consequences of multilingual practices (DeLuca et al., 2020).

While there are many similarities, important differences in how music and language experiences affect cognition and mental health across the life span also exist (Moreno & Bidelman, 2014). There is some evidence for unique effects on cognition as well as shared effects for musical and multilingual experiences in young adults (Bialystok & DePape, 2009; D’Souza et al., 2018), but to date none of this work concerned older adults. Yet, this age group is of particular interest because it is at the older adult life stage that cognitive reserve becomes more pertinent, as cognitive and mental resources are compromised. Although it is impossible to control for all experiences and traits that individuals have and that might be related to mentally healthy aging, it is important to study the interactions of variables and experiences that may drive even marginal cognitive advantages (Leivada et al., 2021; Valian, 2015). Crucially, as it suggested that the wider the spectrum of lifetime experiences, the more cognitive reserve is likely to be enhanced (Krivanek et al., 2021; Stern, 2012, 2021), potential cumulative effects of musical and multilingual experiences need to be examined more closely in a senior population.

The current study aims to investigate whether musical and multilingual experiences are related to cognitive performance and subjective well-being in older adults. It examines whether there are differences between these experiences and, crucially, assesses their cumulative effects. We expect that better executive functioning, measured using the Ruff Figural Fluency Test (RFFT), and emotional well-being, measured using the Positive and Negative Affect Schedule (PANAS), are related to at least one such an experience compared to no such life experience. We assume that engaging in multiple complex life experiences is more challenging and stimulating than a single experience. It is therefore hypothesized that combined experiences have a stronger relationship with cognitive and mental health. Studying unique, shared, and cumulative effects of lifelong engagement to complex skill learning in the domain of music and multilingualism will provide insights into healthy aging and how complex life experiences relate to more healthy life years.

## Method

### Study Sample

This study used data from the *Lifelines Cohort Study*, a multidisciplinary prospective population-based cohort study examining in a unique three-generation design the health and health-related behaviors of 167,729 persons living in the North of the Netherlands (described in Scholtens et al., 2015). It employs a broad range of investigative procedures in assessing the biomedical, sociodemographic, behavioral, physical, and psychological factors which contribute to the health and disease of the general population, with a special focus on multimorbidity and complex genetics. Recruitment and baseline measurements of the *Lifelines* study started in 2006 and ended in 2013. Written informed consent was obtained from all participants. The *Lifelines Cohort Study* is conducted according to the principles of the Declaration of Helsinki and approved by the ethics committee of the University Medical Center Groningen, The Netherlands.

### Language and Music Background Questionnaire

*Lifelines* participants aged 65 years or older in December 2019 (approximately 20,500 participants) were approached via e-mail through the *Lifelines* infrastructure and asked to complete a questionnaire tapping their musical and multilingual backgrounds and experiences. A total of 11,335 participants responded and completed the questionnaire between December 2019 and April 2020.

The musical background and experience questionnaire was based on the questionnaire used in Gooding et al. (2014). Seven questions prompted information pertaining to (1) musical (including singing) experiences at any life stage [yes/no], (2) musical engagement experiences as part of a group or solitary activity [yes/no], (3) with or without instruction [yes/no], (4) age of onset playing a musical instrument or singing, (5) life years during which musical activities were most actively practiced [<18, 18–30, 31–60, >60 years old], (6) the intensity [>1, 1, <1 hr a week], and duration of musical engagement during the most active period [5-point Likert scale ranging from every day to more than once a month], and (7) current activities playing an instrument or singing [yes/no].

The multilingual background and experiences questionnaire contained 16 questions and was based on the questionnaire used in Pot et al. (2018). It tapped information including (1) mastered/learned languages and dialects (dialects are treated as languages), (2) self-reported receptive and productive proficiency in these languages [scale 1–10], (3) the age of, and (4) contexts in which these languages were learned [with/without schooling], (5) self-perceived multilingual status [multilingual yes/no], (6) current multilingual language use in daily life [yes/no], (7) frequency of current or past multilingual language use [5-point Likert scale ranging from every day to more than once a month], (8) multilingual switching behavior [3-point Likert scale ranging from no switching to switching often], and (9) social networks and settings in which multiple languages are used [at home, at work, at society/club, business, other].

### Cognitive and Mental Health Variables

The RFFT (Ruff et al., 1987) was included as a measure of cognitive health, and the Dutch version of the PANAS (Watson et al., 1988) as a measure of emotional well-being. The RFFT and the PANAS were administered during the baseline measurement (between 2006 and 2013). Of all Lifelines measures, these two were selected as measures of cognitive and mental health because they were administered at the same time, while this was not the case for other measures included in the Lifelines database. Although there was a lag of several years between administration of the RFFT and PANAS and the time when the musical and multilingual backgrounds and experience questionnaire were sent out, we believe that this is justified because exposure to musical and/or multilingual experiences is assumed to be relatively stable over this time span. Only 1% of the participants started playing a musical instrument and 4% stopped using multiple languages between the moment of assessment of the RFFT/PANAS and the musical and multilingual questionnaire.

The RFFT was used as a broad measure of executive functioning as it relies on many different cognitive processes, among which initiation, planning (strategies), cognitive flexibility, and divergent reasoning (Kuiper et al., 2017). The RFFT is a paper and pencil test where participants—within the time span of 60 s—draw as many unique designs as possible on a sheet of paper with 35 dots arranged in seven rows and five columns, by connecting the dots in different patterns. This is repeated five times, each time using a sheet with a different stimulus pattern. Performance on the RFFT is expressed by the total number of unique designs (i.e., the sum of all five parts), with scores ranging between 0 and 175. The RFFT is sensitive to small differences in cognitive performance of older adults, has shown good test–retest reliability (Foster et al., 2005; Ruff et al., 1987), and can discriminate between different educational levels and ages (Izaks et al., 2011).

The PANAS is a questionnaire that consists of 10 items (phrased as adjectives) to measure positive affect (e.g., enthusiastic, inspired) and 10 items measuring negative affect (e.g., distressed, irritable). In the *Lifelines* study, affect was measured using the PANAS over the past 4 weeks (i.e., how participants felt over the past 4 weeks). Participants responded to the items on a 5-point Likert scale ranging from 1 (*not at all*) to 5 (*very much*). The total score for each subscale was computed by summing the items from each subscale, resulting in scores ranging from 10 to 50 for each subscale. Higher scores on the positive and negative scales indicated higher positive and negative affect, respectively.

### Statistical Analyses

**Figure 1.**
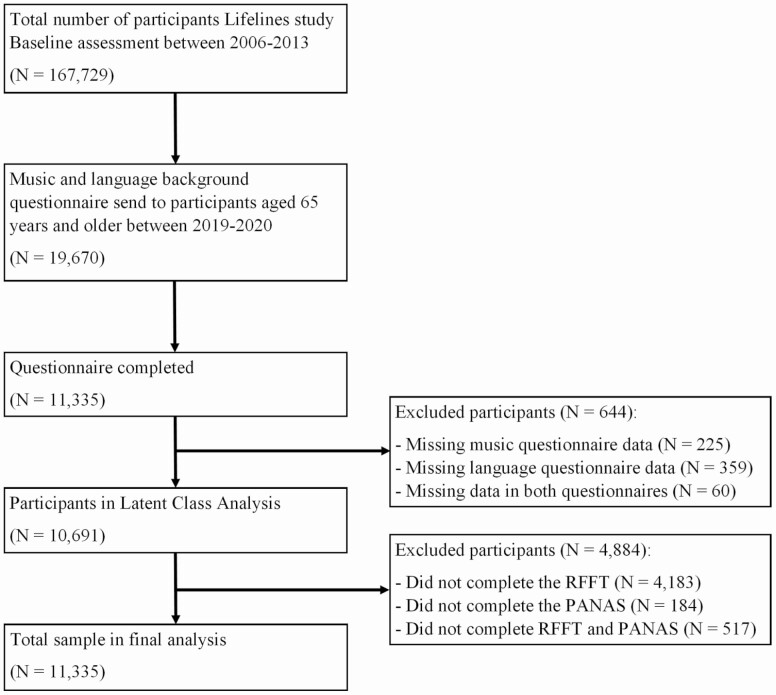
Flow chart of data collection. PANAS = Positive and Negative Affect Schedule; RFFT = Ruff Figural Fluency Test.

Participants who completed the musical and multilingual experience and background questionnaire (*N* = 10,691, see Figure 1) were categorized into subgroups according to their various musical and multilingual experiences using latent class analysis (LCA). The exact description on how this was performed can be found in Supplementary Materials. Descriptive statistics were calculated on the basis of the emergent subgroups with respect to demographics (age, level of education, and income level) and musical and multilingual background and experiences.

*Lifelines* data on the RFFT and PANAS were complete for 5,807 out of the total number of participants (*N* = 10,691) categorized using LCA (see Figure 1). LCA was not performed over this smaller sample, but over the larger sample to include more individuals and therefore more experiences, independently of any potential bias that may have resulted from not having completed the RFFT or PANAS. Descriptive statistics were also performed on the final sample of 5,807 participants for age (at baseline and at time of administration of the musical and multilingual experience questionnaire), sex, level of education, and monthly net household income level (all at baseline). Inferential statistics were used to describe subgroup comparability.

Educational level was categorized as low (primary education, and lower vocational or junior secondary education), middle (secondary vocational and senior secondary education), or high (higher vocational education and university education). Net income level was categorized as low (<2,000 euros/month), middle (2,000–3,000 euros/month), or high (>3,000 euros/month).

As a next step, data (*N* = 5,807) were subjected to a multinomial logistic regression analysis. Group, as determined using LCA, served as the dependent categorical variable. The number of unique designs on the RFFT, positive affect, and negative affect served as independent variables to predict group. Age at baseline, level of education, and income level (as indicators of socioeconomic status [SES]) were added as covariates to the multinomial logistic regression model. Multinomial logistic regression was performed using the “multinom” function from the nnet package in R (Venables & Ripley, 2002). The regression coefficients from the model were converted into odds ratios by exponentiating them. The odds ratios are an unstandardized effect size statistic: the larger the odds ratio, the larger the effect. As the R package does not include *p*-value calculation for the regression coefficients, *p*-values were calculated using Wald tests (following Liang et al., 2020).

## Results

### Latent Classes (Subgroups)

Four latent classes formed the optimal solution (see Supplementary Materials) to describe the total sample (*N* = 10,691). Classes were clearly defined into groups with and without musical experience (Table 1). Multilingualism was considered as a scale ranging from low to high multilingual load based on an individual combination of the number of learned languages, current multilingual language use, and frequency of multilingual language use. The different classes could then be characterized as (1) no musical experience and low multilingualism (*nonmusical, low multilingual [nMlM]*, *N* = 1,441, 13%); (2) no musical experience but high multilingual (*nonmusical, high multilingual [nMhM]*, *N* = 3,148, 29%); (3) musical experience but low multilingualism (*musical, non-multilingual [MlM]*, *N* = 1,430, 13%); and (4) musical experience and high multilingualism (*musical, high multilingual [MhM]*, *N* = 4,672, 44%). A description of the different classes and their musical and multilingual experience can be found in Supplementary Materials.

**Table 1.** Descriptive Statistics on Demographics and Musical and Language Experiences Per Subgroup

				Subgroup			
				1—nMlM Nonmusical non-multilingual	2—nMhM Nonmusical multilingual	3—MlM Musical non-multilingual	4—MhM Musical multilingual
Total per subgroup	*N* (%)			1,441 (14%)	3,148 (29%)	1,430 (13%)	4,672 (44%)
Age at baseline	Mean (*SD*)			63.05 (4.71)	62.22 (4.65)	63.12 (4.65)	62.56 (4.63)
Age at questionnaire	Mean (*SD*)			71.85 (4.62)	71.04 (4.54)	71.90 (4.56)	71.37 (4.55)
Income level	*N* (%)	1	Low	222 (31%)	444 (25%)	223 (29%)	578 (22%)
		2	Middle	220 (31%)	573 (33%)	221 (29%)	883 (34%)
		3	High	130 (18%)	485 (28%)	185 (24%)	770 (30%)
Educational attainment	*N* (%)	1	Low	443 (62%)	784 (45%)	357 (47%)	919 (36%)
		2	Middle	158 (22%)	513 (39%)	187 (24%)	630 (24%)
		3	High	95 (13%)	405 (23%)	195 (25%)	968 (38%)
Ever played musical instrument or singing	Median			No	No	Yes	Yes
	*N* (%)		Yes	0	0	1,430 (100%)	4,672 (100%)
			No	1,441 (100%)	3,148 (100%)	0	0
Current play musical instrument or singing	Median			NA	NA	No	No
	*N* (%)		Yes	0	0	409 (29%)	1,715 (37%)
			No	0	0	1,021 (71%)	2,957 (63%)
Frequency playing musical instrument or singing	Median			NA	NA	3	2
	*N* (%)	1	Every day	0	0	179 (13%)	730 (16%)
		2	More than once a week	0	0	534 (37%)	2,120 (45%)
		3	3–5 times a month	0	0	657 (46%)	1,659 (35%)
		4	1–2 times a month	0	0	48 (3%)	119 (3%)
		5	Less than once a month	0	0	12 (1%)	44 (1%)
Number of learned languages	Median			2	4	3	5
	*N* (%)		1	471 (33%)	68 (2%)	278 (19%)	66 (1%)
			2	302 (21%)	598 (19%)	242 (17%)	626 (13%)
			3	203 (14%)	595 (19%)	212 (15%)	662 (14%)
			4	285 (20%)	665 (21%)	379 (27%)	918 (20%)
			5	156 (11%)	873 (28%)	247 (17%)	1,530 (33%)
			6	<25 (2%)	256 (8%)	54 (4%)	595 (13%)
			7	<10 (1%)	93 (3%)	18 (1%)	275 (6%)
Current multilingual language use	Median			no	yes	No	Yes
	*N* (%)		Yes	24 (2%)	2,917 (93%)	14 (1%)	4,407 (94%)
			No	1,417 (98%)	231 (5%)	1,416 (99%)	265 (6%)
Frequency of multilingual language use	Median			5	2	5	2
	*N* (%)	1	Every day	<25 (2%)	1,066 (34%)	<10 (1%)	1,616 (35%)
		2	More than once a week	<10 (1%)	893 (28%)	<25 (2%)	1,392 (30%)
		3	Once a week	44 (3%)	407 (13%)	71 (5%)	543 (12%)
		4	Once a month	114 (8%)	294 (9%)	146 (10%)	449 (10%)
		5	Less than once a month	1,258 (87%)	488 (16%)	1,187 (83%)	672 (14%)

*Notes*: Percentages present the percentage of participants per subgroup. Numbers and percentages do not always add up to the total numbers of participants (100%) in the subgroups for the variables age, income level, and educational attainment due to missing data. Percentages may not always add up to 100% due to rounding. To avoid identifying individuals and numbers under 10, in case of an *N* of 10 or less for a specific response category, the specific *N* is not mentioned for that response category and the closest consecutive response category. nMlM = nonmusical, low multilingual; nMhM = nonmusical, high multilingual; MlM = musical, low multilingual; MhM = musical, high multilingual.

### Sample Characteristics

The sample for which PANAS and RFFT data were available (*N* = 5,807) is approximately 54% of the total sample used in LCA (*N* = 10,691) to create the subgroups. This percentage is reflected within the four subgroups, except for subgroup 3 (1, *nMlM*: *N* = 713 [49%]; 2, *nMhM*: *N* = 1,749 [55%]; 3, *MlM*: N = 765 [12%]; 4, *MhM*: *N* = 2,580 [55%]).

Of the final sample of 5,807 participants, 53% was female (*N* = 3,075) and the average age of the sample was 71.40 (*SD* = 4.55, range 65.0–93.8) years at the time of administration of the musical and multilingual background and experience questionnaire (administered between December 2019 and April 2020), and 62.59 (*SD* = 4.65, range 52.3–86.3) years at the time of administering the RFFT and PANAS (baseline measurement; administered between 2006 and 2013). An analysis of variance test of variance showed that the subgroups differed significantly from each other in age at baseline (*F*(3,5770) = 9.24, *p* < .001), and age at the moment of completing the music and language questionnaire (*F*(3,5803) = 9.16, *p* < .001). Subgroup 3 (*MlM*) was the oldest at both assessment waves, whereas subgroup 2 (*nMhM*) was the youngest. Furthermore, Kruskal–Wallis tests determined that the subgroups also differed in baseline level of education (*H*(3) = 233.8, *p* < .001) and baseline income level (*H*(3) = 52.8, *p* < .001). Subgroup 4 (*MhM*) was more highly educated and had a higher income level than the other subgroups, whereas subgroup 1 (*nMlM*) showed a comparatively lower level of education and lower income level.

### Cognitive and Mental Health

Mean RFFT and PANAS scores are listed in Table 2 for all subgroups. Multinomial logistic regression was performed three times, once with subgroup 1 (*nMlM*), once with subgroup 4 (*MhM*), and once with subgroup 2 (*nMhM*) as the reference category. All other groups are compared against the reference group, allowing a direct comparison between two groups. Which subgroup is taken as reference is important regarding the comparisons that can be made between the groups, but makes no difference for the calculated coefficients, probabilities, or significances (Kwak & Clayton-Matthews, 2002). The regression analyses were tested against a conservative Bonferroni-adjusted alpha level of 0.0167 (= 0.05/3). Results of these analyses are presented in Table 3. All models were controlled for age, level of education, and income level.

**Table 2.** Descriptive Statistics on RFFT and PANAS Scores

Variables		1—nMlM Nonmusical non-multilingual (*N* = 713)	2—nMhM Nonmusical multilingual (*N* = 1,749)	3—MlM Musical non-multilingual (*N* = 765)	4—MhM Musical multilingual (*N* = 2,580)
RRFT unique designs	Mean (*SD*)	64.30 (18.79)	69.58 (20.14)	69.10 (20.47)	73.48 (21.40)
	Range	1–130	1–132	10–133	1–149
PANAS positive affect	Mean (*SD*)	34.92 (3.87)	35.21 (3.91)	34.97 (3.98)	35.66 (3.80)
	Range	18–48	19–48	15–48	17–49
PANAS negative affect	Mean (*SD*)	20.25 (4.88)	19.79 (4.88)	20.53 (4.96)	20.31 (4.97)
	Range	10–37	10–42	10–37	10–43

*Notes*: PANAS = Positive and Negative Affect Schedule; RFFT = Ruff Figural Fluency Test; *SD* = standard deviation; nMlM = nonmusical, low multilingual;  nMhM = nonmusical, high multilingual; MlM = musical, low multilingual; MhM = musical, high multilingual.

**Table 3.** Results of the Multinomial Logistic Regression Analyses

		Reference	Subgroup	Odds ratio	*p*	95% CI
RFFT unique designs	Model 1	1 (nMlM)	2 (nMhM)	1.006	.014*	1.00–1.01
		1 (nMlM)	3 (MlM)	1.007	.033	1.00–1.01
		1 (nMlM)	4 (MhM)	1.014	<.001**	1.01–1.02
	Model 2	4 (MhM)	1 (nMlM)	0.986	<.001**	0.98–0.99
		4 (MhM)	2 (nMhM)	0.992	<.001**	0.99–1.00
		4 (MhM)	3 (MlM)	0.992	.001*	0.99–1.00
	Model 3	2 (nMhM)	3 (MlM)	1.000	.965	1.00–1.01
Positive affect	Model 1	1 (nMlM)	2 (nMhM)	1.012	.372	0.99–1.04
		1 (nMlM)	3 (MlM)	1.004	.805	0.97–1.03
		1 (nMlM)	4 (MhM)	1.049	<.001**	1.02–1.08
	Model 2	4 (MhM)	1 (nMlM)	0.953	<.001**	0.93–0.98
		4 (MhM)	2 (nMhM)	0.963	<.001**	0.95–0.98
		4 (MhM)	3 (MlM)	0.956	<.001**	0.93–0.98
	Model 3	2 (nMhM)	3 (MlM)	0.992	.532	0.97–1.02
Negative affect	Model 1	1 (nMlM)	2 (nMhM)	0.987	.210	0.97–1.01
		1 (nMlM)	3 (MlM)	1.018	.135	0.99–1.04
		1 (nMlM)	4 (MhM)	1.029	.004*	1.01–1.05
	Model 2	4 (MhM)	1 (nMlM)	0.971	.004*	0.95–0.99
		4 (MhM)	2 (nMhM)	0.959	<.001**	0.94–0.97
		4 (MhM)	3 (MlM)	0.989	.249	0.97–1.01
	Model 3	2 (nMhM)	3 (MlM)	1.032	<.001**	1.01–1.05
Age	Model 1	1 (nMlM)	2 (nMhM)	0.968	.003	0.95–0.99
		1 (nMlM)	3 (MlM)	1.010	.430	0.99–1.04
		1 (nMlM)	4 (MhM)	0.988	.231	0.97–1.01
	Model 2	4 (MhM)	1 (nMlM)	1.013	.231	0.99–1.03
		4 (MhM)	2 (nMhM)	0.981	.012*	0.97–1.00
		4 (MhM)	3 (MlM)	1.023	.024	1.00–1.04
	Model 3	2 (nMhM)	3 (MlM)	1.043	<.001**	1.02–1.06
Education High	Model 1	1 (nMlM)	2 (nMhM)	2.032	<.001**	1.51–2.73
		1 (nMlM)	3 (MlM)	2.473	<.001**	1.77–3.45
		1 (nMlM)	4 (MhM)	3.965	<.001**	2.99–5.25
	Model 2	4 (MhM)	1 (nMlM)	0.252	<.001**	0.19–0.33
		4 (MhM)	2 (nMhM)	0.512	<.001**	0.43–0.61
		4 (MhM)	3 (MlM)	0.624	<.001**	0.49–0.79
	Model 3	2 (nMhM)	3 (MlM)	1.217	.127	0.95–1.57
Education Middle	Model 1	1 (nMlM)	2 (nMhM)	1.692	<.001**	1.33–2.15
		1 (nMlM)	3 (MlM)	1.457	<.010*	1.09–1.94
		1 (nMlM)	4 (MhM)	1.649	<.001**	1.30–2.09
	Model 2	4 (MhM)	1 (nMlM)	0.607	<.001**	0.48–0.77
		4 (MhM)	2 (nMhM)	1.026	.760	0.87–1.21
		4 (MhM)	3 (MlM)	0.884	.293	0.70–1.11
	Model 3	2 (nMhM)	3 (MlM)	0.861	.212	0.68–1.09
Income Middle	Model 1	1 (nMlM)	2 (nMhM)	1.166	.193	0.93–1.47
		1 (nMlM)	3 (MlM)	0.899	.443	0.69–1.18
		1 (nMlM)	4 (MhM)	1.300	.022	1.04–1.63
	Model 2	4 (MhM)	1 (nMlM)	0.769	.022	0.61–0.96
		4 (MhM)	2 (nMhM)	0.897	.205	0.76–1.06
		4 (MhM)	3 (MlM)	0.692	<.001**	0.55–0.86
	Model 3	2 (nMhM)	3 (MlM)	0.771	.027	0.61–0.97
Income High	Model 1	1 (nMlM)	2 (nMhM)	1.333	.039	1.01–1.75
		1 (nMlM)	3 (MlM)	0.998	.991	0.73–1.37
		1 (nMlM)	4 (MhM)	1.232	.125	0.94–1.61
	Model 2	4 (MhM)	1 (nMlM)	0.812	.125	0.62–1.06
		4 (MhM)	2 (nMhM)	1.082	.405	0.90–1.30
		4 (MhM)	3 (MlM)	0.810	.095	0.63–1.04
	Model 3	2 (nMhM)	3 (MlM)	0.749	.028	0.58–0.97

*Notes*: Multinomial logistic regression analysis was performed three times. First with subgroup 1 (nMlM) as reference group, next with subgroup 4 (MhM) as reference group, and with subgroup 2 (nMhM) as reference group. Regression models were corrected for age, level of education, and income level. CI = confidence interval; RFFT = Ruff Figural Fluency Test; nMlM = nonmusical, low multilingual; nMhM = nonmusical, high multilingual; MlM = musical, low multilingual; MhM = musical, high multilingual.

*Significant at the level of .0167, corrected for multiple testing using Bonferroni.

**Significant at the level of .001.

The multinomial logistic regression with subgroup 1 (*nMlM*) as the reference category allowed assessment of the effect of having a musical and/or multilingual compared to no or little experience. The analysis showed that compared to subgroup 1 (*nMlM*) the high-multilingual experience subgroups (subgroups 2 [*nMhM*] and 4 [*MhM*]) were significantly more likely to show better executive functioning (Model 1, Table 3), while controlling for age, level of education, and income level. The largest odds ratio, and thus the largest difference, was found between subgroup 1 and subgroup 4. Executive functioning uniquely related to group membership, in addition to the significant relation between level of education and group membership. The same analysis was performed with subgroup 4 (*MhM*) as the reference category to test the effect of having both musical and high-multilingual life experiences compared to only one experience. Results of Model 2 (Table 3) showed that individuals with better RFFT performance were significantly more likely to fall into the reference group (subgroup 4 [*MhM*]) as compared to subgroups 2 (*nMhM*) and 3 (*MlM*). Additionally, repeating the analysis with subgroup 2 (*nMM*) as the reference category allowed assessment of differences between having only musical experience compared to having only multilingual experience. No significant difference between subgroups 2 and 3 was found on the RFFT score (Model 3, Table 3).

For affect, higher levels of positive affect were significantly more likely for the reference group (subgroup 4 [*MhM*]) than for all other subgroups (Model 2, Table 3). No significant effects for positive affect were found when comparing subgroups 1, 2, and 3 (Models 1 and 3, Table 3). Furthermore, individuals with higher negative affect levels were also significantly more likely in subgroup 4 as compared to subgroup 1 (*nMlM*) and subgroup 2 (*nMhM*), but not to subgroup 3 (*MlM*, Model 2, Table 3). Higher levels of negative affect were furthermore more likely attested in subgroup 3 (*MlM*) compared to the reference subgroup 2 (*nMhM*, Model 3, Table 3). Effects were found while controlling for age, level of education, and income level.

## Discussion

This study examined whether musical and multilingual life experiences are related to cognition and emotional well-being and whether there are cumulative effects of both experiences in a large population-based sample of older adults in the northern Netherlands.

The single experience groups (subgroups 2 and 3) demonstrated similar levels of executive functioning and positive affect. Therefore, in contrast to previous work in young adults (Alain et al., 2018; Bialystok & DePape, 2009; D’Souza et al., 2018; Moradzadeh et al., 2015), we did not find support for a difference in cognitive performance between musical and multilingual experiences, which is consistent with other work (Bialystok & DePape, 2009; Moreno et al., 2014; Schroeder et al., 2016). Results did indicate a difference having no musical and low-multilingual experience (i.e., no experience, subgroup 1) and having multilingual experience: the high-multilingual group (subgroup 2) was more likely show better executive functioning. The group with only musical experience also outperformed the group with no experiences, but this result did not survive multiple comparison correction. Therefore, with regard to cognitive performance, multilingualism does not seem to be more important than musical experience, but having either experience is better than having neither.

Crucially, as complex as musical and multilingual life experiences are in and by themselves, in individuals with both life experiences, we see the highest cognitive performance scores: the group with both musical and high-multilingual experience showed the highest RFFT scores. The idea that accumulated complex life experiences contribute more strongly than a single experience is underlined by the results of this study on positive affect as a measure of subjective well-being: the group with both experiences demonstrated more positive feelings compared to the group with only one or no experience. Having both experiences relates more strongly to positive affect and cognitive effects compared to having either experience alone or neither experience.

Interestingly, the effect for negative affect was more variable. The group with both musical and high-multilingual experience demonstrated higher negative affect than all other groups, except for the group with only musical experience. While this may seem contradictory, it is possible that having multiple life experiences, and thus a more varied palette of activities, leads to a more varied emotional palette as well. More activities may relate to more life engagement, which may relate to greater enjoyment, but also greater risk for involvement in disappointments (Isaacowitz & Smith, 2003). Alternatively, people with higher levels of negative affect may engage in activities to reduce negative affectivity. This could explain the relation between high negative affect and practicing music, as music experience has been found to reduce negative affect (Groarke & Hogan, 2019; Seinfeld et al., 2013).

Cognitive performance and complex experiences thus seem to be related, but our study design does not permit for causal inference. Speculating on the direction of causation, individuals with higher levels of cognitive functioning and emotional well-being might be more likely to engage in and sustain in practicing music and multilingualism. However, one usually does not speak multiple languages due to a certain talent or interest, but this is often simply a function of circumstances or one’s living situation (Bialystok et al., 2012). Alternatively, cognitive performance may emerge from complex life experiences. Musical and multilingual experiences both constitute complex and sustained life experiences that rely on several cognitive functions and multiple underlying brain networks. It could therefore be argued that the complex process of (lifelong) adapting to a new environment that could contribute to cognition and well-being in older adults. This conclusion is in line with others suggesting that complex experiences (Park, 2019; Valenzuela & Sachdev, 2006) and a broad spectrum of experiences (Krivanek et al., 2021; Stern, 2012, 2021) may contribute to cognitive performance in later life where cognitive reserve effects emerge. However, this is the first study to relate the combination of musical and multilingual experiences to cognitive performance in older adults specifically.

Different complex, sustained, and intense life experiences may involve different brain networks, in turn leading to general improvements across cognitive tasks but may also be more pronounced for some tasks than for others, depending on the type of practice (music vs language). Indeed, some studies have found no differences between people with musical or multilingual experiences at the behavioral level but did find differences in brain networks involved (Alain et al., 2018; Moreno & Bidelman, 2014). Although there is overlap and transfer between the two experiences (Asaridou & McQueen, 2013), musical and multilingual experiences stimulate and shape the brain in different ways (Alain et al., 2018; Moreno & Bidelman, 2014). For example, musical training has been shown to be positively associated with inferior frontal cortex and parahippocampal volumes, in addition to posterior cingulate cortex volume, insula and medial orbitofrontal cortex, areas important for memory, emotion regulation, and motivation (Chaddock-Heyman et al., 2021). Multilingualism, on the other hand, has been associated with increased gray matter density in regions implicated in executive control, including the dorsolateral prefrontal cortex, caudate nucleus, and anterior cingulate cortex (Hayakawa & Marian, 2019). Future research would do well to not only explore how multiple complex life experiences relate to task performance but to add an examination of underlying brain networks in addition to behavioral paradigms.

The (combined) effect of musical and multilingual experiences may uniquely apply to later phases of life which could explain earlier null findings. Compared to young adults (aged 30 years or younger), life experiences in older adults (55 years and older) have built up over more years. Factors such as the duration and intensity of the experience may therefore be of great importance. The call for a focus on the intensity of a life experience is in line with current views on the effects of multilingualism (Gullifer & Titone, 2020; Marian & Hayakawa, 2021; Pot et al., 2018). Although we did not further investigate the link between experience intensity and cognition and mental health, future research would do well to tackle this. We furthermore recommend performing a similar study to ours in an older population or a population of older adults with clinical levels of cognitive decline. Life experiences that aid in preserving cognitive functioning and well-being could be even stronger in these populations.

Importantly, we established a relation between life experiences and cognition and affect on the basis of a large population-based sample. Moreover, all results were controlled for age, level of education, and income level (as indicator for SES), while all other variables varied freely within the groups, decreasing the risk of biases. Better executive functioning appears to significantly relate to having multiple complex life experience in addition to the significant relation between life experiences and level of education. This further demonstrates, and reinforces our suggestion, that it is precisely the multiplicity of experiences that likely builds cognitive reserve. Furthermore, while the effect sizes are small, the narrow confidence intervals indicate precise population estimates and therefore reliable evidence. Perhaps the fact that the effects are small explains why findings from earlier studies with smaller samples have produced mixed results.

A number of limitations underlie the current study. First, other experiences or individual traits (which were not measured in the current study) may also play a role. Someone who has multiple complex life experiences may be characterized by certain traits that in fact drive healthy aging (Valian, 2015). It is complex, virtually impossible, to truly isolate experiences that attribute an effect on cognitive reserve to any single experience. It has shown to be already quite complex to characterize the multilingual experience itself that relates to another limitation. The questions from the questionnaire and the way in which they were asked and could be answered allowed for inconsistent answers about someone’s multilingualism. Additionally, and relatedly, a problem in the existing literature is that different cognitive tasks and language measures are used, making it difficult to compare studies and formulate clear predictions. A third important limitation related to the measures of cognition and effect is that these were only momentary assessments and were administered at a different time than the experience questionnaires. Although no major changes are to be expected given the age of the participants, cognition may have changed since the RFFT administration. It was not possible to assess changes in cognition and affect over time and how this change relates to complex life experiences, which would be an interesting direction for future research. It would be interesting for future work to compare how these two life experiences interact with changes in cognition and well-being over time. A final limitation is the limited cognitive characterization of the sample. Factors such as medication use, depression, and MCI were not considered while these factors may have influenced the results. On the other hand, despite the fact that these and other factors varying within the population may lead to substantial interindividual variation, the current study still found a relation between complex life experiences and cognition and emotional well-being.

In conclusion, high executive functioning, positive and negative affect were all related to having complex life experiences. The current study suggests that musical and multilingual experiences are both related to healthy aging. Having either experience is by itself associated with better scores than having neither, but multilingualism is not more important than musical experience, and having both is superior to having either one of the two experiences. A broader spectrum of lifetime experiences is suggested to relate to cognitive reserve. However, although language and music experiences precede the cognitive and affective measures, the cross-sectional design of this study does not allow for causal interpretations.

## Supplementary Material

gbac185_suppl_Supplementary_Material

## References

[CIT0001] Alain, C., Khatamian, Y., He, Y., Lee, Y., Moreno, S., Leung, A. W. S., & Bialystok, E. (2018). Different neural activities support auditory working memory in musicians and bilinguals. Annals of the New York Academy of Sciences, 1423(1), 435–446. doi:10.1111/nyas.13717 29771462

[CIT0002] Alain, C., Moussard, A., Singer, J., Lee, Y., Bidelman, G. M., & Moreno, S. (2019). Music and visual art training modulate brain activity in older adults. Frontiers in Neuroscience, 13, 1–15. doi:10.3389/fnins.2019.00182 30906245 PMC6418041

[CIT0003] Asaridou, S. S., & McQueen, J. M. (2013). Speech and music shape the listening brain: Evidence for shared domain-general mechanisms. Frontiers in Psychology, 4, 1–14. doi:10.3389/fpsyg.2013.00321 23761776 PMC3671174

[CIT0004] Bialystok, E . (2017). The bilingual adaptation: How minds accommodate experience. Psychological Bulletin, 143(3), 233–262. doi:10.1037/bul0000099 28230411 PMC5324728

[CIT0005] Bialystok, E., Craik, F. I. M., & Luk, G. (2012). Bilingualism: Consequences for mind and brain. Trends in Cognitive Sciences, 16(4), 240–249. doi:10.1016/j.tics.2012.03.001 22464592 PMC3322418

[CIT0006] Bialystok, E., & DePape, A.-M. (2009). Musical expertise, bilingualism, and executive functioning. Journal of Experimental Psychology Human Perception and Performance, 35(2), 565–574. doi:10.1037/a0012735 19331508

[CIT0007] Bubbico, G., Chiacchiaretta, P., Parenti, M., di Marco, M., Panara, V., Sepede, G., Ferretti, A., & Perrucci, M. G. (2019). Effects of second language learning on the plastic aging brain: Functional connectivity, cognitive decline, and reorganization. Frontiers in Neuroscience, 13, 423. doi:10.3389/fnins.2019.00423 31156360 PMC6529595

[CIT0008] Bugos, J. A., Perlstein, W. M., McCrae, C. S., Brophy, T. S., & Bedenbaugh, P. H. (2007). Individualized Piano Instruction enhances executive functioning and working memory in older adults. Aging and Mental Health, 11(4), 464–471. doi:10.1080/13607860601086504 17612811

[CIT0009] Chaddock-Heyman, L., Loui, P., Weng, T. B., Weisshappel, R., McAuley, E., & Kramer, A. F. (2021). Musical training and brain volume in older adults. Brain Sciences, 11(1), 50. doi:10.3390/brainsci11010050 33466337 PMC7824792

[CIT0011] D’Souza, A. A., Moradzadeh, L., & Wiseheart, M. (2018). Musical training, bilingualism, and executive function: Working memory and inhibitory control. Cognitive Research: Principles and Implications, 3(1), 11. doi:10.1186/s41235-018-0095-6 29670934 PMC5893660

[CIT0056] Dause, T., & Kirby, E. (2019). Aging gracefully: Social engagement joins exercise and enrichment as a key lifestyle factor in resistance to age-related cognitive decline. Neural Regeneration Research, 14(1), 39. doi:10.4103/1673-5374.243698 30531067 PMC6262997

[CIT0012] DeLuca, V., Rothman, J., Bialystok, E., & Pliatsikas, C. (2020). Duration and extent of bilingual experience modulate neurocognitive outcomes. NeuroImage, 204, 116222. doi:10.1016/j.neuroimage.2019.116222 31557543

[CIT0013] Dorris, J. L., Neely, S., Terhorst, L., VonVille, H. M., & Rodakowski, J. (2021). Effects of music participation for mild cognitive impairment and dementia: A systematic review and meta-analysis. Journal of the American Geriatrics Society, 69(9), 2659–2667. doi:10.1111/jgs.17208 34008208 PMC8440389

[CIT0010] European Commission Directorate-General for Economic and Financial Affairs. (2020). The 2021 Ageing Report. doi:10.2765/733565

[CIT0014] Foster, P. S., Williamson, J. B., & Harrison, D. W. (2005). The Ruff Figural Fluency Test: Heightened right frontal lobe delta activity as a function of performance. Archives of Clinical Neuropsychology, 20(4), 427–434. doi:10.1016/j.acn.2004.09.010 15896557

[CIT0057] Fratiglioni, L., Paillard-Borg, S., & Winblad, B. (2004). An active and socially integrated lifestyle in late life might protect against dementia. The Lancet Neurology, 3(6), 346–353. doi:10.1016/S1474-4422(04)00767-7 15157849

[CIT0015] Gooding, L. F., Abner, E. L., Jicha, G. A., Kryscio, R. J., & Schmitt, F. A. (2014). Musical training and late-life cognition. American Journal of Alzheimer’s Disease & Other Dementiasr, 29(4), 333–343. doi:10.1177/1533317513517048 PMC4074275 24375575

[CIT0016] Groarke, J. M., & Hogan, M. J. (2019). Listening to self-chosen music regulates induced negative affect for both younger and older adults. PLoS One, 14(6), e0218017. doi:10.1371/journal.pone.0218017 31170224 PMC6553776

[CIT0017] Gullifer, J. W., & Titone, D. (2020). Characterizing the social diversity of bilingualism using language entropy. Bilingualism: Language and Cognition, 23(2), 283–294. doi:10.1017/S1366728919000026

[CIT0018] Hanna-Pladdy, B., & MacKay, A. (2011). The relation between instrumental musical activity and cognitive aging. Neuropsychology, 25(3), 378–386. doi:10.1037/a0021895 21463047 PMC4354683

[CIT0019] Hayakawa, S., & Marian, V. (2019). Consequences of multilingualism for neural architecture. Behavioral and Brain Functions, 15(1), 6. doi:10.1186/s12993-019-0157-z 30909931 PMC6432751

[CIT0020] Isaacowitz, D. M., & Smith, J. (2003). Positive and negative affect in very old age. The Journals of Gerontology, Series B: Psychological Sciences and Social Sciences, 58(3), 143–152. doi:10.1093/geronb/58.3.p143 12730307

[CIT0021] Izaks, G. J., Joosten, H., Koerts, J., Gansevoort, R. T., & Slaets, J. P. (2011). Reference data for the Ruff Figural Fluency Test stratified by age and educational level. PLoS One, 6(2), e17045. doi:10.1371/journal.pone.0017045 21347325 PMC3037396

[CIT0022] Kramer, A. F., Bherer, L., Colcombe, S. J., Dong, W., & Greenough, W. T. (2004). Environmental influences on cognitive and brain plasticity during aging. The Journals of Gerontology, Series A: Biological Sciences and Medical Sciences, 59(9), M940–M957. doi:10.1093/gerona/59.9.m940 15472160

[CIT0023] Krivanek, T. J., Gale, S. A., McFeeley, B. M., Nicastri, C. M., & Daffner, K. R. (2021). Promoting successful cognitive aging: A ten-year update. Journal of Alzheimer’s Disease, 81(3), 871–920. doi:10.3233/jad-201462 PMC8293659 33935078

[CIT0024] Kroll, J. F., & Bialystok, E. (2013). Understanding the consequences of bilingualism for language processing and cognition. Journal of Cognitive Psychology, 25(5), 497–514. doi:10.1080/20445911.2013.799170 PMC3820916 24223260

[CIT0025] Kuiper, J. S., Oude Voshaar, R. C., Verhoeven, F. E. A., Zuidema, S. U., & Smidt, N. (2017). Comparison of cognitive functioning as measured by the Ruff Figural Fluency Test and the CogState computerized battery within the LifeLines Cohort Study. BMC Psychology, 5, 15. doi:10.1186/s40359-017-0185-0 28494817 PMC5427615

[CIT0058] Kwak, C., & Clayton-Matthews, A. (2002). Multinomial logistic regression. Nursing Research, 51(6), 404–410. doi:10.1097/00006199-200211000-00009 12464761

[CIT0026] Leivada, E., Westergaard, M., Duñabeitia, J. A., & Rothman, J. (2021). On the phantom-like appearance of bilingualism effects on neurocognition: (How) should we proceed? Bilingualism: Language and Cognition, 24(1), 197–210. doi:10.1017/S1366728920000358

[CIT0027] Li, P., Legault, J., & Litcofsky, K. A. (2014). Neuroplasticity as a function of second language learning: Anatomical changes in the human brain. Cortex, 58, 301–324. doi:10.1016/j.cortex.2014.05.001 24996640

[CIT0028] Liang, J., Bi, G., & Zhan, C. (2020). Multinomial and ordinal logistic regression analyses with multi-categorical variables using R. Annals of Translational Medicine, 8(16), 982. doi:10.21037/atm-2020-57 32953782 PMC7475459

[CIT0029] Machón, M., Larrañaga, I., Dorronsoro, M., Vrotsou, K., & Vergara, I. (2017). Health-related quality of life and associated factors in functionally independent older people. BMC Geriatrics, 17, 19. doi:10.1186/s12877-016-0410-3 28088178 PMC5237562

[CIT0030] Mansky, R., Marzel, A., Orav, E. J., Chocano-Bedoya, P. O., Grünheid, P., Mattle, M., Freystätter, G., Stähelin, H. B., Egli, A., & Bischoff-Ferrari, H. A. (2020). Playing a musical instrument is associated with slower cognitive decline in community-dwelling older adults. Aging Clinical and Experimental Research, 32, 1577–1584. doi:10.1007/s40520-020-01472-9 32144734

[CIT0031] Marian, V., & Hayakawa, S. (2021). Measuring bilingualism: The quest for a “bilingualism quotient.” Applied Psycholinguistics, 42(2), 527–548. doi:10.1017/s0142716420000533 34054162 PMC8158058

[CIT0032] Moradzadeh, L., Blumenthal, G., & Wiseheart, M. (2015). Musical training, bilingualism, and executive function: A closer look at task switching and dual-task performance. Cognitive Science, 39(5), 992–1020. doi:10.1111/cogs.12183 25289704

[CIT0033] Moreno, S., & Bidelman, G. M. (2014). Examining neural plasticity and cognitive benefit through the unique lens of musical training. Hearing Research, 308, 84–97. doi:10.1016/j.heares.2013.09.012 24079993

[CIT0034] Moreno, S., Wodniecka, Z., Tays, W., Alain, C., & Bialystok, E. (2014). Inhibitory control in bilinguals and musicians: Event related potential (ERP) evidence for experience-specific effects. PLoS One, 9(4), e94169. doi:10.1371/journal.pone.0094169 24743321 PMC3990547

[CIT0035] Mukadam, N., Sommerlad, A., & Livingston, G. (2017). The relationship of bilingualism compared to monolingualism to the risk of cognitive decline or dementia: A systematic review and meta-analysis. Journal of Alzheimer’s Disease, 58, 45–54. doi:10.3233/jad-170131 28387680

[CIT0036] Olszewska, A. M., Gaca, M., Herman, A. M., Jednoróg, K., & Marchewka, A. (2021). How musical training shapes the adult brain: Predispositions and neuroplasticity. Frontiers in Neuroscience, 15, 204. doi:10.3389/fnins.2021.630829 PMC7987793 33776638

[CIT0037] Park, D. C . (2019). Cognitive ability in old age is predetermined by age 20 y. Proceedings of the National Academy of Sciences of the United States of America, 116(6), 1832–1833. doi:10.1073/pnas.1821142116 30700549 PMC6369757

[CIT0038] Pfenninger, S. E., & Polz, S. (2018). Foreign language learning in the third age: A pilot feasibility study on cognitive, socio-affective and linguistic drivers and benefits in relation to previous bilingualism of the learner. Journal of the European Second Language Association, 2(1), 1–13. doi:10.22599/jesla.36

[CIT0039] Pot, A., Keijzer, M., & de Bot, K. (2018). Intensity of multilingual language use predicts cognitive performance in some multilingual older adults. Brain Sciences, 8(5), 92. doi:10.3390/brainsci8050092 29783764 PMC5977083

[CIT0040] Román-Caballero, R., Arnedo, M., Triviño, M., & Lupiáñez, J. (2018). Musical practice as an enhancer of cognitive function in healthy aging—A systematic review and meta-analysis. PLoS One, 13(11), e0207957. doi:10.1371/journal.pone.0207957 30481227 PMC6258526

[CIT0041] Ruff, R. M., Light, R. H., & Evans, R. W. (1987). The Ruff Figural Fluency Test: A normative study with adults. Developmental Neuropsychology, 3(1), 37–51. doi:10.1080/87565648709540362

[CIT0042] Scheibe, S., & Carstensen, L. L. (2010). Emotional aging: Recent findings and future trends. The Journals of Gerontology, Series B: Psychological Sciences and Social Sciences, 65(2), 135–144. doi:10.1093/geronb/gbp132 PMC2821944 20054013

[CIT0043] Schneider, C. E., Hunter, E. G., & Bardach, S. H. (2019). Potential cognitive benefits from playing music among cognitively intact older adults: A scoping review. Journal of Applied Gerontology, 38(12), 1763–1783. doi:10.1177/0733464817751198 29361873

[CIT0044] Scholtens, S., Smidt, N., Swertz, M. A., Bakker, S. J. L., Dotinga, A., Vonk, J. M., Van Dijk, F., van Zon, S. K. R., Wijmenga, C., Wolffenbuttel, B. H. R., & Stolk, R. P. (2015). Cohort Profile: LifeLines, a three-generation cohort study and biobank. International Journal of Epidemiology, 44(4), 1172–1180. doi:10.1093/ije/dyu229 25502107

[CIT0045] Schroeder, S. R., Marian, V., Shook, A., & Bartolotti, J. (2016). Bilingualism and musicianship enhance cognitive control. Neural Plasticity, 2016, 4058620. doi:10.1155/2016/4058620 26819764 PMC4706931

[CIT0046] Seinfeld, S., Figueroa, H., Ortiz-Gil, J., & Sanchez-Vives, M. V. (2013). Effects of music learning and piano practice on cognitive function, mood and quality of life in older adults. Frontiers in Psychology, 4, 810. doi:10.3389/fpsyg.2013.00810 24198804 PMC3814522

[CIT0047] Song, S., Stern, Y., & Gu, Y. (2022). Modifiable lifestyle factors and cognitive reserve: A systematic review of current evidence. Ageing Research Reviews, 74, 101551. doi:10.1016/j.arr.2021.101551 34952208 PMC8794051

[CIT0048] Stern, Y . (2012). Cognitive reserve in ageing and Alzheimer’s disease. The Lancet Neurology, 11(11), 1006–1012. doi:10.1016/S1474-4422(12)70191-6 23079557 PMC3507991

[CIT0049] Stern, Y . (2021). How can cognitive reserve promote cognitive and neurobehavioral health? Archives of Clinical Neuropsychology, 36(7), 1291–1295. doi:10.1093/arclin/acab049 34651645 PMC8517622

[CIT0050] Valenzuela, M. J., & Sachdev, P. (2006). Brain reserve and dementia: A systematic review. Psychological Medicine, 36(4), 441–454. doi:10.1017/S0033291705006264 16207391

[CIT0051] Valian, V . (2015). Bilingualism and cognition. Bilingualism: Language and Cognition, 18(1), 3–24. doi:10.1017/S1366728914000522

[CIT0052] van den Noort, M., Vermeire, K., Bosch, P., Staudte, H., Krajenbrink, T., Jaswetz, L., Struys, E., Yeo, S., Barisch, P., Perriard, B., Lee, S.-H., & Lim, S. (2019). A systematic review on the possible relationship between bilingualism, cognitive decline, and the onset of dementia. Behavioral Sciences, 9(7), 81. doi:10.3390/bs9070081 31340609 PMC6680432

[CIT0053] Venables, W. N., & Ripley, B. D. (2002). Modern applied statistics with S (4th ed.). Springer. doi:10.1007/978-0-387-21706-2

[CIT0054] Watson, C. W., Manly, J. J., & Zahodne, L. B. (2016). Does bilingualism protect against cognitive aging? Linguistic Approaches to Bilingualism, 6(5), 590–604. doi:10.1075/lab.15043.wat 30505373 PMC6263976

[CIT0055] Watson, D., Clark, L. A., & Tellegen, A. (1988). Development and validation of brief measures of positive and negative affect: The PANAS scales. Journal of Personality and Social Psychology, 54(6), 1063–1070. doi:10.1037//0022-3514.54.6.1063 3397865

