# Supplementary material for: Musical and Multilingual Experience Are Related to Healthy Aging: Better Some Than None But Even Better Together
Source: J Gerontol B Psychol Sci Soc Sci. 2022 Dec 1;78(4):609–19. doi: 10.1093/geronb/gbac185 (PMC10066740; doi:10.1093/geronb/gbac185)
Supplement: gbac185_suppl_Supplementary_Material [file gbac185_suppl_supplementary_material.docx]

**Latent Class Analysis (LCA)**

**Introduction**

A prominent and topical issue in multilingualism research is that researchers are increasingly recognizing that multilingualism is an experience that is situated on a continuum rather than constituting a categorical variable. This can be extended to musical experiences. Comparing groups of individuals with or without life experiences is considered unsuitable because it masks the variability within the groups. In addition, to create groups, an arbitrary boundary must often be imposed resulting in different criteria for each study. Latent Class Analysis (LCA) overcomes these issues: rather than splitting the data into groups based on – somewhat arbitrarily – predetermined criteria, groups were made according to the patterns of individual answers to the musical and multilingual background and experience questionnaire.

**Method**

Based on an exploratory LCA, data of three questions on musical experience and three questions on multilingual experience and background served as input for the LCA: 1) musical (including singing) experience at any life stage [yes/no], 2) frequency of musical engagement during the most active period [5-point Likert scale ranging from every day to more than once a month], 3) current activities playing an instrument or singing [yes/no], 4) the number of mastered/learned languages and dialects [1-7], 5) current multilingual language use in daily life [yes/no], 6) frequency of current or past multilingual language use [5-point Likert scale ranging from every day to more than once a month]. There is one score for each of these questions for each participant and this data served as the input for the LCA. Participants with incomplete data on these six questions were removed prior to the LCA. This was the case for 644 subjects, leaving 10,691 subjects as input for the LCA (see Figure 1).

To find the best fitting model, following guidelines by Weller et al. (2020), the LCA model was built multiple times with a varying number of classes, ranging from two to eight classes. Model selection was based on the value of Bayesian Information Criterion (BIC) and sample-size adjusted BIC (aBIC), where a lower value suggests a better fit (Muthen & Muthen, 2000). All models were re-estimated 10 times to find the optimal maximum likelihood solution (Linzer & Lewis, 2011). The model that best fit the data was selected and subsequently used to assign all 10,691 participants to their predicted subgroup for further analysis. The final model was furthermore described in terms of entropy as an estimate of how accurately the model defines classes (Linzer & Lewis, 2011). Although no consensus for cutoff value exists for the evaluation of entropy, values closer to 1.0 indicate better classification quality and values greater than 0.80 are indicative of adequate classification quality (Brose et al., 2014; Jung & Wickrama, 2008).

**Results**

To decide on the number of classes, LCA was performed multiple times on all available data (10,691 participants). A careful examination of the models led to a selection of a four-class model solution as the best fit for the data, as evidenced by the BIC, and aBIC values (see Supplementary Table 1). This four-class model had an entropy value of 0.90, reflecting a clear delineation of classes.

| Supplementary Table 1  *Fit of Latent Class Analysis models* | | | |  |
| --- | --- | --- | --- | --- |
| **Latent class numbers** | **Likelihood ratio G^2^** | **BIC** | **aBIC** | |
| 2 | 5746.7 | 119558.5 | 119434.6 | |
| 3 | 3270.2 | 117264.5 | 117077.0 | |
| 4 | 899.0 | 115078.7 | 114827.6 | |
| 5 | 824.6 | 115189.7 | 117150.0 | |
| 6 | 728.6 | 115361.5 | 114900.9 | |
| 7 | 654.0 | 115444.3 | 114948.2 | |
| 8 | 624.9 | 115546.9 | 115040.9 | |
| *Note.* BIC = Bayesian Information Criterion, aBIC = sample-size adjusted BIC. *N* = 10,691 | | | | |

## Latent classes (subgroups)

*Musical experience*

Participants in the two musical subgroups (subgroups 3 [*MlM*], and 4 [*MhM*]) had all a musical experience (among which singing) at some point in their lives, but most did not currently actively pursue musical activities (see Table 1). People had started playing an instrument or singing at the mean age of 19.0 years in subgroup 3 (SD = 15.7), and at the mean age of 17.5 years in subgroup 4 (SD = 14.7). The mean age is probably a rather skewed representation given that most participants actively played a musical instrument before the age of 18 (57.2% and 57.8% for subgroup 3 and 4, respectively). A substantial percentage also actively played a musical instrument or sang after the age of 60 (24.6% and 28.0% for subgroup 3 and 4, respectively). Most participants who learned to play a musical instrument did so under the guidance of a music teacher in a one-to-one instructional setting (66.6% in subgroup 3, 71.6% in subgroup 4) and only 26.7% and 33.0% for subgroup 3 and 4, respectively, played an instrument as part of a group or band. In the case of singing, on the other hand, most participants did so as part of a group activity (57.6% in subgroup 3, 60.0% in subgroup 4). The subgroups differed in frequency of playing/singing (see Table 1), while in both subgroups participants were most likely to have played/sung more than one hour a week (62.6% in subgroup 3, 71.2% in subgroup 4).

*Multilingual experiences*

The number of spoken languages varied in all subgroups from one to seven. Supplementary Table 2 presents which languages were mainly spoken and by what percentage of participants in each group.

In the two low-multilingual subgroups (subgroups 1 and 3), more than 70% of the participants reported Dutch as their L1 in contrast to approximately 50% of the participants in the two high-multilingual subgroups (subgroups 2 and 4). Frisian was reported more often as participants’ L1 in subgroups 2 and 4 (23.0% and 23.2%, respectively) compared to approximately 5% in subgroups 1 and 3, and Dutch as L2. Apart from Dutch, in all subgroups the Lower Saxon dialect was also often reported as L1 (subgroup 1: 17.8%, subgroup 2: 21.5%; subgroup 3: 13.8%; subgroup 4: 19.6%). English, German, and French (in this order) were most frequently reported as additional languages in all subgroups. These languages are taught at Dutch schools and are spoken in neighboring countries.

Within the two high-multilingual subgroups (subgroups 2 [*nMhM*] and 4 [*MhM*]), and as opposed to the musical groups, by far most participants reported to still use multiple languages in daily life (see Table 1). Most often, participants reported not to switch between their spoken languages within one situation or setting (49.1% subgroup 2, 46.7% subgroup 4). Instead, different languages were reportedly used in different settings with different interlocutors. Frequent switches between languages within settings was only applicable for 6% and 7% of the sample in subgroups 2 and 4, respectively. Most participants in subgroups 2 and 4 reported to use multiple languages with relatives (57.5% and 57.7%, respectively) and friends (55.6% and 60.0%, respectively), primarily in the home situation (50.0% and 49.5%, respectively). Participants in both subgroups had most commonly learned their first language (L1) without (formal) education, in contrast to additional languages, which were most often reported to have been learned through (formal) education. Apart from the L1, participants generally indicated to be better at understanding a language than speaking it. Participants were best able to speak and understand their L2 (understand: M = 8.61, SD = 1.57 subgroup 2; M = 8.70, SD = 1.51 subgroup 4; speak: M = 8.17, SD = 1.79 subgroup 2; M = 8.25, SD = 1.78 subgroup 4). Not all participants within the high-multilingual subgroups perceived themselves as multilingual, although the majority did: in subgroup 2, 71.3% and in subgroup 4, 77.3% of the participants self-categorized as multilingual.

Supplementary Table 2

*The most reported spoken languages per subgroup and the number (and percentage) of participants per subgroup that reported to speak these languages*

| Reported language  N (%) | 1 – nMlM  nonmusical non multilingual  (N = 1441) | 2 – nMhM  nonmusical  multilingual  (N = 3148) | 3 – MlM musical  non multilingual  (N = 1430) | 4 – MhM musical multilingual  (N = 4672) |
| --- | --- | --- | --- | --- |
| Dutch | 1217 (88%) | 2966 (94%) | 1299 (91%) | 4371 (94%) |
| English | 697 (48%) | 2079 (66%) | 917 (64%) | 3504 (75%) |
| German | 496 (34%) | 1871 (59%) | 731 (51%) | 3229 (69%) |
| French | 348 (24%) | 1349 (43%) | 601 (42%) | 2656 (56%) |
| Lower Saxon | 379 (26%) | 1338 (43%) | 351 (25%) | 1946 (42%) |
| Frisian | 154 (11%) | 1250 (40%) | 213 (15%) | 1949 (42%) |
